# Barriers and facilitators of rehabilitation nursing care for patients with disability in the rehabilitation hospital: A qualitative study

**DOI:** 10.3389/fpubh.2022.931287

**Published:** 2022-08-11

**Authors:** Shima Shirozhan, Narges Arsalani, Sadat Seyed Bagher Maddah, Farahnaz Mohammadi-Shahboulaghi

**Affiliations:** ^1^Nursing Department, University of Social Welfare and Rehabilitation Science, Tehran, Iran; ^2^Iranian Research Center on Aging, Nursing Department, University of Social Welfare and Rehabilitation Sciences, Tehran, Iran

**Keywords:** rehabilitation nursing, inpatient rehabilitation, people with disability, quality of care, nursing practice, rehabilitation team

## Abstract

Nurses play a key role in providing rehabilitation care. In this regard, identifying the factors that affect their practice can be useful in planning to improve the quality of rehabilitation nursing care. This study aims to explore the experience of nurses and members of the rehabilitation team about barriers and facilitators of rehabilitation nursing care of patients with disability in the rehabilitation hospital. This qualitative study was conducted in the main public rehabilitation hospital in Tehran, Iran. Eighteen persons including 12 nurses in clinical and managerial positions, an occupational therapist, a physical medicine specialist, a patient, and an informal caregiver participated in this study. Participants were selected based on purposeful sampling. Data were collected through 18 in-depth semi-structured interviews and analyzed based on qualitative content analysis principles. Three themes were derived from the data analysis, which represented Barriers and facilitators related to nurses (specialized knowledge and skills, psychological status, mentoring, professional communication), barriers and facilitators related to the work environment (nurses' performance evaluation, nursing workforce, comprehensive care facilities, workplace design, specialized unit), barriers and facilitators related to patients and caregivers (patient's participation in nursing care, patient adaptation, efficiency of formal caregivers). The experiences of the rehabilitation team shows that not only nurses, but also the environment, patients, and caregivers can affect the provision of care and change the quality of care. Identifying these factors can help managers, researchers, and clinical nurses to facilitate and improve rehabilitation nursing care by modifying the influencing factors.

## Introduction

Rehabilitation is one of the fundamental services in health care that is provided for a wide range of diseases and health problems at acute, sub-acute, and long-term phases (1). The aim of rehabilitation is to promote, achieve and maintain maximum performance through training and empowerment of people with disabilities to manage their health status and adapt to their conditions (2, 3). The rehabilitation process is based on teamwork. Members of the rehabilitation team, including physicians, nurses, physiotherapists, occupational therapists, and psychotherapists are involved in this process (4, 5). Among the members of the rehabilitation team, nurses play a key role in the rehabilitation process and have several responsibilities in providing rehabilitation care (6). Nursing care prepares patients for the rehabilitation process, leads to the continuation of rehabilitation, achieves desired rehabilitation outcomes, and improves patients' quality of life (7, 8). Due to the inability of patients to perform activities of daily living, nurses help them to meet basic needs (7, 9). In addition to meeting physical needs, nurses support patients in psychological, spiritual, and social dimensions (10, 11). Providing comprehensive rehabilitation nursing care in all dimensions requires some prerequisites. In addition to the nurse's competence in providing care, contextual factors such as environmental characteristics, care equipment, and leadership affect the nurse's ability to provide comprehensive care (12–15). In 2014, Clarke identified some of the factors affecting nursing care by reviewing the literature on stroke rehabilitation. Some of these identified factors such as the clarity of nurses' roles, the interdisciplinary work, and evidence-based practice facilitated nursing care (16). Also, problems such as nursing shortage, focus on routine practice, parallel or separate teamwork, and hierarchical relationships between nurses and therapists prevented the provision of rehabilitation nursing care (16). All these factors affect the provision of holistic nursing care and the safety and quality of care. In addition, considering the importance of nursing care role in the rehabilitation process, barriers and facilitators can affect the achievement of rehabilitation outcomes. They also affect the health status and quality of life of people with disabilities (4, 13, 16, 17). Barriers can reduce nurses' performance due to mental fatigue, dissatisfaction, and burnout (18–21). Despite the importance of recognizing these challenges, few studies have been conducted to identify the factors affecting rehabilitation nursing care. Among these studies, quantitative research is unable to comprehensively explain the affecting factors. Qualitative studies have focused solely on a specific area such as stroke rehabilitation. In addition, most of them have focused on examining the viewpoints of nurses and the experiences of other members of the rehabilitation team in working with rehabilitation nurses have been less considered (13–16). Therefore, this qualitative study was conducted to explore the rich experiences of all members of the rehabilitation team on barriers and facilitators of rehabilitation nursing care for patients with disability in the rehabilitation hospital.

## Materials and methods

### Design

Deep understanding of different people's experiences about human phenomena such as rehabilitation nursing care can be achieved through qualitative research. Content analysis is one of the most widely used techniques in qualitative research, especially in the field of nursing, which aims to provide knowledge and understanding of the phenomenon studied. Since the aim of this study was to understand the experiences of nurses and other members of the rehabilitation team about barriers and facilitators of rehabilitation nursing care, this qualitative study was carried out with a content analysis approach (22–24).

### Participants

The study population consisted of the members of the rehabilitation team in Rofaydeh Rehabilitation hospital in Tehran. Inclusion criteria include a bachelor's degree or higher and 6 months of work experience in a rehabilitation hospital for nurses; experience working with rehabilitation nurses for rehabilitation team members; experience of hospitalization in Rofaydeh rehabilitation hospital for patients and caregivers. Participants were included in the study based on purposeful sampling. the first participant was selected by the research team based on the participant's experience and willingness. Maximum variation in Sampling was performed to obtain a wide range of experiences. Eighteen persons including 12 nurses (eight clinical nurses, two head nurses and one educational supervisor, and one clinical supervisor), an occupational therapist, a physical medicine and rehabilitation specialist, a nurse assistant, a patient with multiple sclerosis with a history of three periods of hospitalization and a caregiver of a patient with stroke participated in this study.

### Data gathering

Data were collected through 18 semi-structured in-depth interviews. Before the meeting, coordination was made about the time and place according to the participants' willingness. Before starting the interview, the necessary explanations about the research were provided and informed written consent was obtained. Based on the interview guide prepared by the research team, interviews started and continued with questions tailored to the role of each participant, for example, clinical nurses were asked that “Please tell me what factors affect the care you provide to patients?” Rehabilitation team members were asked “what factors affect nurses' care provision based on your experience working with nurses?”. The following questions were based on participant responses and the questions were referred differently. At the end of each interview, the interviewer was asked to provide additional information by asking, “Do you want to add anything else?”. The interview continued for as long as the participant wanted and lasted an average of 45–60 min.

### Data analysis

In this study, the data were analyzed using conventional content analysis based on the five stages of Graneheim and Lundman. In the first step, In the first stage, the recorded interviews were transcribed verbatim. The interviews were read several times to understand the whole sense of the interview. The text of the interview was read and divided into meaning units and were abstracted and labeled with a code. During coding and labeling meaning units, the main context and whole sense were considered. The codes were compared based on differences and similarities and sorted into categories. The tentative categories were discussed by the research team and revised. Finally, the underlying meaning, that is the latent content of the categories was formulated into themes (23). Interviews continued until no new categories or relevant themes emerged and the categories evolved in terms of conceptualization (24).

### Trustworthiness and ethical consideration

In order to ensure the trustworthiness of the data based on Lincoln and Guba's evaluation criteria, credibility, dependability or accountability, transferability and confirmability were addressed. In this regard, the researcher was present in the research environment for more than a year to collect qualitative data and tried to gather valid information through prolong engagement in conducting interviews. Participants with maximum diversity were selected in terms of education, work experience, gender, and job position. Through the process of member check, the initial coding of interviews was examined by participants to verify the accuracy of the codes. Also, in order to conduct peer review, three faculty members familiar with qualitative research examined the codes and categories and agreed on interpretations. Clear and distinct descriptions of culture and context, selection and characteristics of participants, data collection, and analysis process were presented (22, 23, 25).

This research is part of a larger study. The present study has been approved by the Ethics Committee of the University of Rehabilitation Sciences and Social Health (Code of ethics: IR.USWR.REC.1400.069) In this study, the researcher adhered to ethical considerations such as confidentiality of participants' names, permission to record interviews, obtaining informed written consent to participate in the study, and the discretion of participants to withdraw from the study at each stage of the study.

## Results

### Participants characteristics

Eighteen persons including 12 nurses, an occupational therapist, a physical medicine and rehabilitation specialist, a nurse assistant, a patient with multiple sclerosis with a history of three periods of hospitalization, and a caregiver of a patient with stroke participated in this study. The nurses participating in the study included 8 clinical nurses, two head nurses, one educational supervisor, and one clinical supervisor. Among the nurses participating in this study, eight worked as clinical nurses, two heads of nursing, one clinical supervisor, and one educational supervisor at Rofaydeh Rehabilitation Hospital. The educational degree of nurses was from undergraduate to doctoral level. five nurses were master's students or graduates of nursing rehabilitation with an average of 2 years of work experience.

Three themes were derived from the data analysis, which represented Barriers and facilitators related to nurses (specialized knowledge and skills, psychological status, mentoring, professional communication), barriers and facilitators related to the work environment (nurses' performance evaluation, nursing workforce, comprehensive care facilities, workplace design, specialized unit), barriers and facilitators related to patients and caregivers (patient's participation in nursing care, patient adaptation, efficiency of formal caregivers).

### Barriers and facilitators related to nurses

This theme suggests that some nurses' issues such as specialized knowledge and skills, psychological status, mentoring, and professional communication can affect the provision of rehabilitation nursing care.

#### Specialized knowledge and skills

This category stated that due to the lack of a comprehensive and special course in the field of rehabilitation nursing in the undergraduate nursing curriculum, nurses do not acquire specialized knowledge and skills in this field and face challenges while working in a rehabilitation hospital. The number of nurses with a master's degree in rehabilitation nursing is limited and these nurses often have little experience working in a rehabilitation hospital. Rehabilitation nursing care is specialized care, and a lack of specialized knowledge and skills prevents the provision of comprehensive and specialized care. A nurse who is a master's student of rehabilitation nursing (p3) stated that:

“At the undergraduate level, I learned a little bit about rehabilitation, but now that I'm studying rehabilitation, my perspective has changed… for example, I noticed that my colleagues didn't educate patients about home modification when they saw that I was training patients about it, they became curious, and I explained it to them”.

In relation to the lack of specialized knowledge of novice nurses, (P6) mentioned this:

“I have a bachelor's degree in nursing, when I came to this hospital, I knew nothing about rehabilitation, caring for people with disabilities was difficult for me because I only knew how to care for patients with general problems such as diabetes or heart diseases”.

The hospital's educational supervisor (p12) stated:

“One of our problems is the small number of nurses with rehabilitation expertise, some of whom are still students... To manage this problem, one of the prerequisites for employment in this hospital is to study the principles of rehabilitation, but it is not enough, because The field of rehabilitation is extensive, and we need to train them constantly to develop their knowledge and improve their skills”.

#### Psychological status

Long-term hospitalization, slow progression and long recovery process of disabled patients, lack of psychological and emotional readiness of nurses to work in rehabilitation units, and difficulty of care are factors that impose a psychological burden on nurses. These psychological stresses affect nurses' performance and prevent them from comprehensively presenting their roles and responsibilities. (P6) in this regard stated:

“a nurse who works in the rehabilitation hospital is under pressure... It's hard to see people with these conditions, Patients' condition affects our work…They always talk to us about their problems and it's too hard to listen to them Because their disease is often incurable, and we can only help them adapt... If something happens to them, even after discharge, we become very sad... they are like our family”.

The psychological burden of care has made nurses feel that they need support, but the emotional and psychological status of nurses is usually ignored. In these conditions, psychological stress increases day by day and reduces their performance. (P7) described:

“To continue working, nurses need to be emotionally strengthened.... One of our colleagues holds motivational classes for patients to strengthen their psychological condition, we also need to attend these kinds of classes…”.

#### Mentoring

The number of nurses with rehabilitation expertise is limited and most of the nurses working in the rehabilitation hospital are general nurses. In this situation, rehabilitation nurses and experienced nurses as a mentor can help and guide their colleagues to provide better care. The presence of experienced nurses in each work shift can help to manage the problems of patients in specific situations. (P4), who has long work experience in a rehabilitation hospital, has explained:

“When someone has the high experience, they know how to act in different situations, and a less experienced colleague can get help if needed”.

p6, who has less experience in working in the rehabilitation hospital, has stated:

“For example, there are some things that we may ignore, but the guidance of our experienced colleague or head nurse helps us to consider more details, and this affects our quality of care a lot, not just doing routine nursing work.... I learned a lot about rehabilitation this way”.

In addition, some nurses have other responsibilities, such as specialized wound care, holding motivational classes for patients, or providing educational content and education in charge. The presence of nurses who have been trained and experienced in a particular field such as wound care can help other nurses in providing specialized care. (P1) who is in charge of education in one of the units stated:

“I am in charge of education in this unit. I help my colleagues provide the best educational content for their patients...If they have questions about educating patients or need more educational resources, I will provide them”.

Regarding the importance of having wound care specialist nurses in facilitating care, (P6) described:

“Some nurses in this hospital are experts in wound care, they teach us how to dress different kinds of wounds, even when they are not in the hospital, we call them, and they guide us. In this situation, we can easily and correctly dress any wound”.

#### Professional communication

Communication of rehabilitation team members with each other is one of the most important requirements of the rehabilitation process. Nurses 'communication with other members of the rehabilitation team helps to be aware of patients' progress in rehabilitation. Also, if any physical or psychological problem occurs in the rehabilitation process, therapists notify nurses and nurses can consider it in providing care. The occupational therapist participating in the study (p17) stated:

“Sometimes patients have a hypotensive episode in the occupational unit, so we call and notify nurses, and when the patient returns to the unit, the nurses assess the patient's hemodynamic condition and take care of him/her, or when the patient complains of shoulder pain, we inform nurses to manage pain by using a sling or something else”.

Communication among nurses is an important factor affecting care. The development of communication leads to the integration of the care process and the provision of comprehensive and safe care. One of the participating nurses (P1) stated:

“We have a cyberspace group if special care like a new orthosis is prescribed to a patient, the head nurse informs us in the group, and everyone knows to consider it... The head nurse usually attends teamwork meetings and informs us of the results in person or online. In general, teamwork meetings are useful because we will be aware of the decisions of the rehabilitation team and the condition of patients so if patients need special care or medical intervention we will follow up”.

Developing communication between patients and nurses leads to better awareness of different physical, psychological, social, and spiritual aspects of patients and providing individualized nursing care. In this regard, the patient participating in the study (p14) has stated:

“In this hospital, we and nurses have a good relationship. We're really like a family so it's easier for us to tell them our problems, they understand us better and treat us according to our situation... I think everyone works better when the communication space is productive and friendly”.

### Barriers and facilitators related to the work environment

This theme suggests that factors such as nurses' performance evaluation, nursing workforce, comprehensive care facilities, workplace design, and the specialized unit can facilitate or hinder the delivery of care.

#### Nurses' performance evaluation

The performance of Nurses is constantly evaluated by supervisors and head nurses. These evaluations focus only on defects while nurses expect that positive aspects of their performance be considered as well. This method of evaluation reduces the motivation of nurses to provide care, causing them to only perform their duties and not try to improve the quality of care. (P4) noted that:

“Each month, supervisors randomly select and review the records of several patients, nursing reports are carefully checked, and head nurses are informed about our mistakes. The focus is only on errors However, If the nurse has written a perfect and complete report, it will not be taken into consideration… Sometimes I wonder why I spend so much time writing a good report in detail, they don't care…”.

Nurses expect different aspects of their care to be seen and receive positive feedback. P5 noted:

“You know, it doesn't matter if someone is responsible, for example, I always check that patients do rehabilitation exercises that the physiotherapist gives them and use their night splints, I supervise patient caregivers to brush patients' teeth, despite all the positive work, I was never encouraged, but if there is the slightest mistake in the nursing report, I will be blamed immediately…”.

Identifying and highlighting the positive points of nurses' performance can encourage them to provide better care. (P4) noted:

“We expect our positive points of performance to be identified and when that doesn't happen, the importance of providing better care is diminished gradually and we will no longer be willing to provide the best care!”.

#### Nursing workforce

One of the barriers to providing care is the nursing shortage that causes compressed work schedules. Nurses have to work more, and they don't have enough time to rest after work shifts. Continuous work and fatigue reduce their ability to provide high-quality care. One of the nurses (P5) pointed out:

“We work overtime while we don't want it… Our working conditions are tough, and overtime makes us tired too much, we always feel tired because we can't get enough rest, we will no longer be able to think about comprehensive care”.

In addition, most shifts are 18 h or 12 h to compensate for nursing shortage. One of the nurses (P4) described:

“18-h shifts are too long, most of my shifts are 18 h, I come to the hospital from 13:00 and until 8:00 tomorrow and I only have 1 day to rest afterward. This not only increases the chances of errors but also prevents us from thinking about different aspects of rehabilitation.”.

This problem also causes nurses to take care of more patients. Therefore, in addition to a compressed work schedule and long work shifts, the workload is also higher than the standard. This leads to less time to take care of each patient and focus on minimum requirements. One head nurse who is in charge of planning the work schedule (P9), stated:

“Our unit has 19 beds. According to the standards of the Ministry of Health, we have three fewer nurses, so the working hours of our staff are increased, and Each nurse should take care of 10 patients and will not have enough time to meet all patients' needs or even talk to them”.

#### Comprehensive care facilities

Patients with disabilities usually have comorbidities so, need some diagnostic tests or medical consults, that need to be done accordingly. Delays in treatment can make it difficult to provide care and will be challenging for nurses. Besides, Disabled patients are susceptible to acute events such as falling, seizure, or aspiration. In the absence of adequate facilities in these emergent conditions, nurses would be exhausted and cannot provide comprehensive care to their patients. The clinical supervisor participating in the study (P13) stated that:

“We don't have CT scan or MRI in this center, and when a patient needs these tests, he/she should be transferred to another hospital, which can be dangerous for the patient and increase the workload of nurses. During the transfer, one nurse must take care of that patient so his/her colleague must take care of all patients in the unit”.

The process of transferring the critically ill patient to other hospitals is very long. This long process prevents nurses from providing adequate and equal care for all patients. The nurse, who is a graduate of intensive care nursing (p5), noted:

“We don't have an intensive care unit, when a patient gets severely ill, for example, suffering from respiratory distress, we have to focus on him/her …the patient's transfer is time-consuming...In this situation, we can't supervise the rehabilitation program of other patients, we need to prioritize care and provide only the acute and necessary care.”.

#### Workplace design

Some parts of the hospital environment are not suitable for patients with disability. problems such as the long corridors make it difficult to access and monitor the patients properly. Most rooms have three beds, and the number of beds does not fit the room space. In these rooms, nurses have difficulty providing care because the risk of infection transmission is high, and it is impossible to protect patients' privacy and talk to them privately. Noisy and crowded rooms distract the patient during training and reduce the effectiveness and quality of education. Inappropriate design of rooms also makes it difficult to provide care such as dressing changes or intermittent catheterization. Caregiver of the patient participated (p15) in the study described:

“Sometimes when nurses want to talk to my mother privately, they take her to another place, like the hospital yard, because their room has three beds… Sometimes, because the rooms are crowded, we may not understand some parts of their education and ask them to repeat it, It's hard and time-consuming. Nurses get tired”.

An inappropriate environment in critical situations creates more challenges. A participant who had the experience of participating in cardiovascular resuscitation. (P8) stated:

“Imagine that in this small room we want to bring an emergency trolley near the patient, there's no space for ourselves to resuscitate the patient... In this situation, in which every second is important, we should move everything around to find enough space for resuscitation”.

#### Specialized unit

Only patients with similar problems are admitted to specialized units. Therefore, nurses better identify patients' needs, education becomes more specialized, and the safety and quality of care increase. One participant who works in the multiple sclerosis rehabilitation unit and previously worked in non-specialized units (P1) stated:

“In our unit, only patients with MS are admitted... Nurses exactly know what problems and needs patients may have and they (nurses) know how to identify them… Medication errors decreased because patients have similar problems. It is easier to educate them…For example, we know how to treat patients with MS who have certain moods.”.

In these units, similar problems of patients with certain diseases have been identified and protocols have been developed to manage them. These protocols help nurses to be aware of special risks. Therefore, they can anticipate certain risks such as falling, aspiration, or seizure and prevent them. In addition, when these happen, nurses can provide acute care more quickly and accurately. P10 who works in the stroke unit noted:

“In our units, some of our patients are susceptible to seizure, a protocol has been developed for seizure management, so our nurses know how to take care of a convulsing patient...., patients with stroke are also prone to shoulder subluxation and pain, so we assess this problem in all patients and train their caregivers to prevent subluxation... patient safety improves because we take care according to a specific protocol”.

### Barriers and facilitators related to patients and caregivers

This theme indicates that the patient's cooperation with the nurse facilitates the provision of care and the inefficiency of caregivers in performing their duties is an obstacle in the provision of rehabilitation nursing care.

#### Patient's participation in nursing care

Rehabilitation nursing care requires patient participation in rehabilitation exercises, repetition of trained skills, and adherence to treatment. If the patient is not cooperating, the nurse should spend more time repeating rehabilitation and training interventions and not make progress in learning new skills. Also, patient cooperation in observing the principles of safety and health facilitates the provision of safe and quality care. The patient participant noted:

“Well, some patients do not cooperate with the nurses, for example, the nurse asks the patient to take his medication on time, but the patient does not pay attention and the nurse has to go to the room several times and remind the patient to take his medication.”

Considering the importance of patient participation in providing safe care, the caregiver participating in this study stated:

“Sometimes patients don't listen to nurses and do dangerous things. For example, nurses emphasize that they should get help from the caregiver to get out of bed and not to go out on their own, but not to pay attention.”

One of the participating nurses also stated:

“When patients cooperate with us, our work gets better, for example, when I teach the patient how to do CIC and he participates and learns to do it independently, I can teach him more and he will become independent sooner.”

#### Patient adaptation

Patients admitted to rehabilitation services shortly after injury or illness may not yet be able to adapt. In these situations, denial of physical problems or illness causes them to reject the exercises and training provided by nurses. Failure to accept the condition and functional limitations may put patients at risk and make it difficult for the nurse to take care of them. Some patients are stubborn with nurses at this stage and do not pay attention to nurses' care recommendations. The participating nurse working in the brain and spinal cord injury unit (P4) stated:

“It is difficult for the patient to cope with what happened to him, especially the patient who suffers from a spinal cord injury due to an accident and the patient is stubborn in the first days, does not want to listen to the training. We need to spend a lot of time talking to them to be satisfied to take care of ourselves.”

These patients also suffer from psychological problems that make them less receptive to rehabilitation nursing care. A nurse working in the multiple sclerosis unit (P1) noted:

“When a patient is diagnosed with MS and hospitalized for treatment and rehabilitation during the attack phase, he or she does not pay attention to the recommended diet or behave aggressively...”

#### Efficiency of formal caregivers

Many patients employ formal caregivers, and this has increased during the COVID-19 pandemic. Although these caregivers are introduced to patients by the hospital, their eligibility for patient care is not assessed. Most of them do not have enough knowledge or experience to care for patients with disability. Therefore, instead of using this time to provide comprehensive care for patients, nurses should spend a lot of time training caregivers and monitoring their work. (P9) described:

“One of our biggest challenges at this hospital is private caregivers (formal caregivers). Instead of spending time caring for the patient, we need to educate the private caregivers while these trainings should have been given to them before … We must constantly monitor their work to prevent them from making mistakes”.

In addition to being incompetent for patient care, they do not cooperate well with nurses. This leads to an increase in the workload of nurses and exhausts them. (P3) stated in this regard:

“It's the duty of the caregiver to help patient to do their exercise but they evade from their responsibilities.... Patients should be taken to rehabilitation units on time, but they don't care… we are too busy to check them all the time, it is annoying that we should remind them everything....”.

Although the incompetence of official caregivers creates many problems for nurses, the relevant authorities are not aware of the importance of their role and do not monitor the performance of formal caregivers. One of the participating head nurses (p10) stated:

“Lack of cooperation of caregivers is a burden on nurses, the others do not care. For example, one of our officials said that we don't care who wants to take care of the patient...., while the caregivers and their performance are very important“.

## Discussion

The aim of this study was to investigate Barriers and facilitators of rehabilitation nursing care for patients with disability in the rehabilitation hospital. The findings indicated that some of these factors are related to nurses, patients, and caregivers and some are related to the work environment. Specialized knowledge and skills, psychological status, mentoring, and professional communication are barriers and facilitators factors. The need for specialized knowledge and skills to provide rehabilitation nursing care can be a barrier to providing nursing care. Because of the shortage of nurses with rehabilitation expertise, general nurses with undergraduate education or nurses with other specialties provide care in the rehabilitation hospital. These nurses have not received adequate rehabilitation training and have no special skills in providing rehabilitation care. Previous studies have consistently shown that one of the challenges of rehabilitation nursing care is inadequate training of nurses and a lack of specialized knowledge in the field of rehabilitation (16, 26). The knowledge and skills of nurses affect their performance in providing care, their relationship with patients, and the rehabilitation process (27). In the absence of sufficient knowledge and non-specialized skills, rehabilitation care is not fully provided, participation of nurses in the rehabilitation process is limited, achieving optimal results becomes difficult, and the risk of injury increases (16, 26, 28). According to the findings of this study, in such situations where the number of nurses with rehabilitation expertise is limited, guidance from rehabilitation nurse specialists, nurses with other specialties such as wound care, or experienced nurses are facilitators of rehabilitation care. In accordance with these results, evidence in stroke rehabilitation shows that assisting clinical nurses by advanced professional nurses plays a key role in better understanding comprehensive care and leads to improved care (15).

As a facilitating factor related to nurses, the professional communication among nurses, nurses with other members of the rehabilitation team and nurses with the patients and their caregivers lead to a better understanding of the patient's condition and rehabilitation process. As a result, this improves the performance of all members of the rehabilitation team especially nurses. In line with these findings, previous studies have also shown that communication and teamwork of nurses with each other, as well as other rehabilitation team members, are important facilitators in providing care, increasing the quality and safety of care, and reducing missed nursing care (4, 14, 16, 29).

The findings also indicate that working in rehabilitation units imposes psychological stress on nurses. Because of the close and long relationship between nurses and patients with disability, nurses are faced with patients' problems, and this puts nurses under pressure. The results of other studies also indicate that working in rehabilitation units is very challenging and has a great psychological burden on the members of the rehabilitation team because they are in contact with patients who have experienced unpleasant events, some suffer from pain and they have various physical, psychological, familial, or social obstacles in the path of rehabilitation. In such situations, rehabilitation nurses experience very high psychological stress which leads to emotional exhaustion (21, 30–32). These work-related psychological problems affect the quality of care and, if continued, cause burnout of nurses, which negatively affects the provision of care (33).

The results of this study also indicate that nurses' performance evaluation, nursing workforce, comprehensive care facilities, workplace design, and specialized unit are barriers and facilitators related to the work environment. Based on the findings, the lack of an accurate method of evaluating nurses' performance, focusing solely on weaknesses and deficiencies, and ignoring the positive points of nurses' performance have made them unmotivated in providing care. This can cause nurses to only meet minimum standards and not try to improve care. Evidence also suggests that a lack of encouragement for nurses causes a lack of motivation, affects their performance, and causes less attention to their responsibilities (34). This also leads to reduced responsibility, neglecting patient needs, lack of follow-up, and repeated missed nursing care (35). This situation along with high psychological pressure and heavy workload can cause burnout in nurses (33, 34).

Shortage of the nursing workforce as a barrier increases the working hours and the workload of nurses. In general, caring for patients with disabilities is heavy and difficult and rehabilitation nurses manage disability and associated diseases, meet basic needs, help to perform activities of daily living, and promote the mental health and social status of patients (7, 8, 16). Providing care in this wide range is harder despite the shortage of nursing staff and this prevents comprehensive rehabilitation nursing care. In addition, a high workload can reduce the quality of care and patient outcomes and increase the occurrence of undesirable events such as falls and infections (16, 18, 36).

In addition, due to the lack of comprehensive facilities and resident physicians, nurses face challenges in providing care when acute problems occur. Therefore, the long process of transferring patients for diagnostic tests or intensive care facilities attracts most of the nurses' attention to critical patients and reduces attention to other patients. It is not uncommon for disabled patients to have acute care needs and may need to be transferred to an acute care facility. Therefore, rehabilitation units should be close to acute care facilities and rapid and uninterrupted transfers must be available (37, 38). It is also advisable to equip rehabilitation hospitals with some acute care facilities, such as CT scan imaging so that some problems can be managed without transferring patients to other centers. All these factors affect patient care and can improve the quality of care and outcomes (37–39).

The workplace design for providing care has also created challenges for nurses. Problems such as lack of space prevent nurses from providing education in a calm environment, protecting the privacy and private dialogue with patients, observing hygiene principles and infection control, and acute care. In confirming these results, evidence suggests that the environment affects nurses' performance and can improve the efficiency, effectiveness, and safety of care (12). Other studies also show that environmental barriers affect the relationship between nurse and patient. Single bedrooms can somewhat resolve privacy and infection control, and the patient's distraction in providing education (12, 40). Participants in this study also stated that the design of units has given them less access to patients' rooms. Problems such as long ways to access patients can negatively affect nurses' readiness to provide care and cause dissatisfaction (20).

The results of this study show that some characteristics of the care environment such as specialized units facilitate professional care. In this situation, nursing care and patient education are provided in a specialized manner, patients' problems are well identified, and managed, and safer care is provided. Previous studies also show that the expertise of nurses, specialized nursing care, specific rehabilitation interventions, and provision of rehabilitation care in specialized units lead to better care and achieving more favorable outcomes for patients (38, 41).

Barriers and facilitators related to patients and caregivers were patient participation in nursing care, patient adaptation, and efficiency of official caregivers. Findings indicated that patient participation in rehabilitation nursing care facilitates care delivery. Evidence also indicated that patient participation is one of the most important and fundamental factors and prerequisites for nursing care and the rehabilitation process (42–44). Patients' participation in nursing care facilitates the provision of care, improves nurses' performance, and achieves desired outcomes for patients (42, 43, 45–48). Findings from the literature review show that one of the ways to increase patient participation is to improve nurse-patient communication (43, 49, 50). The professional relationship between nurse and patient is another facilitating factor of rehabilitation nursing care in this study. Therefore, paying attention to improving professional communication can improve synergy by facilitating patient participation to facilitate the provision of rehabilitation care.

The findings of this study indicated that patients' adaptation to their situation facilitates the provision of rehabilitation nursing care. Sister Callista Roy, a nursing theorist, has proposed an adaptation model through which nurses assist patients in achieving adaptation, in other words, nurses facilitate patient adaptation (51–53). However, the authors of this study did not find evidence as to whether the patient's adaptation to their condition facilitates the provision of nursing care.

Another barrier to care is the inefficiency of formal caregivers. The incompetence of formal caregivers forces the nurse to train and prepare them for care and constantly supervise their work. Evidence also suggests that the unpreparedness of caregivers is one of the barriers to rehabilitation care (38). Other studies indicate that there is no specific framework for training and supporting caregivers, causing nurses to spend time to training and preparing them for care to compensate for this shortage. This increases their workload and prevents them from providing rehabilitation care (12, 39–41).

Regarding our results and previous evidence, we state that managers, researchers, and clinical nurses can play an important role in strengthening the facilitator and removing barriers. Nursing managers can improve nurses' professional knowledge, skills, and professional communication by holding training courses with different topics such as rehabilitation principles, communication, and teamwork skills. Proper workplace design for provision of rehabilitation care, equipping and specializing the care units and standard workforce allocation are other tasks that can be done by managers to facilitate rehabilitation care. Clinical nurses' efforts to gain more knowledge and skills and improve communication and collaboration with other members of the rehabilitation team can help them provide better care. Nursing researchers can design and implement interventions in future research to improve nurses' psychological status, increase participation, patient adaptation and efficiency of formal caregivers.

## Conclusion

The results of this study indicated that there are several factors in facilitating or inhibiting the provision of rehabilitation nursing care, some of which are related to nurses, patients and caregivers, and the work environment. Barriers can negatively affect the quality and safety of care, prevent comprehensive care, increase missed nursing care, and cause exhaustion and burnout in nurses. Facilitating factors also promote professional care and provide comprehensive care. Considering the impact of barriers and facilitators on the quality and safety of care, recognizing barriers and facilitators, and planning to eliminate or strengthen them are very important. The results of this study remind us that achieving this goal depends on improving nurses' situation, paying attention to patients and their caregivers, and resolving work environment shortcomings. this finding can help managers, researchers, and clinical nurses to modify these factors to improve rehabilitation nursing care. Considering this study was conducted in the inpatient rehabilitation hospital, it is suggested that future studies be performed in different settings.

## Data availability statement

The original contributions presented in the study are included in the article/supplementary material, further inquiries can be directed to the corresponding author.

## Ethics statement

The studies involving human participants were reviewed and approved by IR.USWR.REC.1400.069. The patients/participants provided their written informed consent to participate in this study.

## Author contributions

SSh conducted interviews and qualitative analysis. All authors designed the study, drafted the manuscript, reviewed the data, read, and approved the manuscript.

## Conflict of interest

The authors declare that the research was conducted in the absence of any commercial or financial relationships that could be construed as a potential conflict of interest.

## Publisher's note

All claims expressed in this article are solely those of the authors and do not necessarily represent those of their affiliated organizations, or those of the publisher, the editors and the reviewers. Any product that may be evaluated in this article, or claim that may be made by its manufacturer, is not guaranteed or endorsed by the publisher.

## References

[1] Bickenbach J. The world report on disability. Disabil Soc. (2011) 26:655–8. 10.1080/09687599.2011.589198

[2] Wade DT. What is Rehabilitation? An Empirical Investigation Leading to an Evidence-Based Description. London, England: SAGE Publications Sage UK. (2020). p. 571–83. 10.1177/0269215520905112 PMC7350200 32037876

[3] World Health Organization. Rehabilitation in Health Systems. Villars-sous-Yens: World Health Organization (2017).

[4] Franz S Muser J Thielhorn U Wallesch CW Behrens J. Inter-professional communication and interaction in the neurological rehabilitation team: a literature review. Disabil Rehabil. (2020) 42:1607–15. 10.1080/09638288.2018.1528634 30457016

[5] Paxino J Denniston C Woodward-Kron R Molloy E. Communication in interprofessional rehabilitation teams: a scoping review. Disabil Rehabil. (2020) 2020:1–17. 10.1080/09638288.2020.1836271 33096000

[6] Gutenbrunner C Stievano A Nugraha B Stewart D Catton H. Nursing–a core element of rehabilitation. Int Nurs Rev. (2021) 69:13–19. 10.1111/inr.12661 33506550

[7] Çol BK Purut HP. Nursing in Rehabilitation Process. Psychol Res. (2018) 8:168–72. 10.17265/2159-5542/2018.04.00530009089

[8] Koç A. Rehabilitation nursing: applications for rehabilitation nursing. Int J Caring Sci. (2012) 5:80.34978776

[9] Kearney PM Lever S. Rehabilitation nursing: invisible and underappreciated therapy. Int J Ther Rehabil. (2010) 2010:394–5. 10.12968/ijtr.2010.17.8.49278

[10] Gutenbrunner C Stievano A Stewart D Catton H Nugraha B. Role of nursing in rehabilitation. Journal of Rehabilitation Medicine-Clinical Communications. (2021) 4. 10.2340/20030711-1000061 34276905 PMC8215228

[11] Aadal L Angel S Langhorn L Pedersen BB Dreyer P. Nursing roles and functions addressing relatives during in-hospital rehabilitation following stroke. Care needs and involvement. Scand J Caring Sci. (2018) 32:871–9. 10.1111/scs.12518 28869654

[12] Lipson-Smith R Pflaumer L Elf M Blaschke S-M Davis A White M. Built environments for inpatient stroke rehabilitation services and care: a systematic literature review. BMJ Open. (2021) 11:e050247. 10.1136/bmjopen-2021-050247 34353805 PMC8344318

[13] Pryor J Walker A O'Connell B Worrall-Carter L. Opting in and opting out: A grounded theory of nursing's contribution to inpatient rehabilitation. Clin Rehabil. (2009) 23:1124–35. 10.1177/0269215509343233 19906766

[14] Kieft RA de Brouwer BB Francke AL Delnoij DM. How nurses and their work environment affect patient experiences of the quality of care: a qualitative study. BMC Health Serv Res. (2014) 14:1–10. 10.1186/1472-6963-14-249 24923663 PMC4064111

[15] Burton CR Fisher A Green TL. The organizational context of nursing care in stroke units: a case study approach. Int J Nurs Stud. (2009) 46:86–95. 10.1016/j.ijnurstu.2008.08.001 18801481

[16] Clarke DJ. Nursing practice in stroke rehabilitation: systematic review and meta-ethnography. J Clin Nurs. (2014) 23:1201–26. 10.1111/jocn.12334 24102924

[17] Meng X Chen X Liu Z Zhou L. Nursing practice in stroke rehabilitation: Perspectives from multi-disciplinary healthcare professionals. Nurs Health Sci. (2020) 22:28–37. 10.1111/nhs.12641 31418173

[18] Harvey C Thompson S Otis E Willis E. Nurses' views on workload, care rationing and work environments. J Nurs Manag. (2020) 28:912–8. 10.1111/jonm.13019 32255223

[19] Danielis M Fantini M Sbrugnera S Colaetta T Maestra MR Mesaglio M. Missed nursing care in a long-term rehabilitation setting: findings from a cross-sectional study. Contemporary Nurse. (2022) 2022:1–32. 10.1080/10376178.2022.2029515 35023449

[20] Kalisch BJ Aebersold M. Interruptions and multitasking in nursing care. Jt Comm J Qual Patient Saf. (2010) 36:126–32. 10.1016/S1553-7250(10)36021-1 20235414

[21] Destiani W Mediawati AS Permana RH. The mental workload of nurses in the role of nursing care providers. J Nursing Care. (2020) 3.

[22] Hsieh H-F Shannon SE. Three approaches to qualitative content analysis. Qual Health Res. (2005) 15:1277−88. 10.1177/1049732305276687 16204405

[23] Graneheim UH Lundman B. Qualitative content analysis in nursing research: concepts, procedures and measures to achieve trustworthiness. Nurse Educ Today. (2004) 24:105–12. 10.1016/j.nedt.2003.10.001 14769454

[24] Speziale HS Streubert HJ Carpenter DR. Qualitative Research in Nursing: Advancing the Humanistic Imperative. Philadelphia, PA: Lippincott Williams & Wilkins (2011).

[25] Lincoln YS Guba EG. Naturalistic Inquiry. San Francisco, CA: Sage (1985).

[26] Booth J Hillier VF Waters KR Davidson I. Effects of a stroke rehabilitation education programme for nurses. J Adv Nurs. (2005) 49:465–73. 10.1111/j.1365-2648.2004.03319.x 15713178

[27] Loft MI Poulsen I Esbensen BA Iversen HK Mathiesen LL Martinsen B. Nurses' and nurse assistants' beliefs, attitudes and actions related to role and function in an inpatient stroke rehabilitation unit—a qualitative study. J Clin Nurs. (2017) 26:4905–14. 10.1111/jocn.13972 28722777

[28] Pound P Ebrahim S. Rhetoric and reality in stroke patient care. Soc Sci Med. (2000) 51:1437–46. 10.1016/S0277-9536(00)00040-X 11077948

[29] Kalisch BJ Lee KH. The impact of teamwork on missed nursing care. Nurs Outlook. (2010) 58:233−41. 10.1016/j.outlook.2010.06.004 20934078

[30] Koerner M. Mental strain among staff at medical rehabilitation clinics in Germany. GMS Psycho-Social-Med. (2011) 8. 10.3205/psm000070 21289992 PMC3028660

[31] McCracken LM Yang S-Y. A contextual cognitive-behavioral analysis of rehabilitation workers' health and well-being: Influences of acceptance, mindfulness, and values-based action. Rehabil Psychol. (2008) 53:479. 10.1037/a0012854

[32] Tay WY Earnest A Tan SY Ng MJM. Prevalence of burnout among nurses in a community hospital in Singapore: a cross-sectional study. Proc Singapore Healthc. (2014) 23:93–9. 10.1177/201010581402300202

[33] Alimoglu MK Donmez L. Daylight exposure and the other predictors of burnout among nurses in a University Hospital. Int J Nurs Stud. (2005) 42:549–55. 10.1016/j.ijnurstu.2004.09.001 15921986

[34] Wihardja H Hariyati RTS Gayatri D. Analysis of factors related to the mental workload of nurses during interaction through nursing care in the intensive care unit. Enfermería Clínica. (2019) 29:262–9. 10.1016/j.enfcli.2019.06.002

[35] Dehghan-Nayeri N Ghaffari F Shali M. Exploring Iranian nurses' experiences of missed nursing care: a qualitative study: a threat to patient and nurses' health. Med J Islam Repub Iran. (2015) 29:276. 26793667 PMC4715394

[36] Racy S Davidson PM Peeler A Hager DN Street L Koirala B. A review of inpatient nursing workload measures. J Clin Nurs. (2021) 30:1799–809. 10.1111/jocn.15676 33503306

[37] Lipson-Smith R Zeeman H Bernhardt J. What's in a building? A descriptive survey of adult inpatient rehabilitation facility buildings in Victoria, Australia. Arch Rehabil Res Clin Transl. (2020) 2:100040. 10.1016/j.arrct.2020.100040 33543069 PMC7853350

[38] Katrak PH Black D Peeva V. Stroke rehabilitation in Australia in a freestanding inpatient rehabilitation unit compared with a unit located in an acute care hospital. PM&R. (2011) 3:716–22. 10.1016/j.pmrj.2011.04.011 21871415

[39] Lipson-Smith R Churilov L Newton C Zeeman H Bernhardt J. A framework for designing inpatient stroke rehabilitation facilities: a new approach using interdisciplinary value-focused thinking. HERDl. (2019) 12:142–58. 10.1177/1937586719831450 30799632 PMC6745610

[40] O'Halloran R Grohn B Worrall L. Environmental factors that influence communication for patients with a communication disability in acute hospital stroke units: a qualitative metasynthesis. Arch Phys Med Rehabil. (2012) 93:S77–85. 10.1016/j.apmr.2011.06.039 22119075

[41] Collaboration SUT. Organised inpatient (stroke unit) care for stroke. Syst Rev. (2013) 2013. 10.1002/14651858.CD000197.pub3 24026639 PMC6474318

[42] Sahlsten MJ Larsson IE Sjöström B Plos KA. Nurse strategies for optimising patient participation in nursing care. Scand J Caring Sci. (2009) 23:490–7. 10.1111/j.1471-6712.2008.00649.x 19552793

[43] Nilsson M From I Lindwall L. The significance of patient participation in nursing care–a concept analysis. Scand J Caring Sci. (2019) 33:244–51. 10.1111/scs.12609 30070390

[44] Lindberg J Kreuter M Taft C Person L. Patient participation in care and rehabilitation from the perspective of patients with spinal cord injury. Spinal Cord. (2013) 51:834–7. 10.1038/sc.2013.97 23999110

[45] Nordgren S Fridlund B. Patients' perceptions of self-determination as expressed in the context of care. J Adv Nurs. (2001) 35:117–25. 10.1046/j.1365-2648.2001.01828.x 11442689

[46] Henderson S. Power imbalance between nurses and patients: a potential inhibitor of partnership in care. J Clin Nurs. (2003) 12:501–8. 10.1046/j.1365-2702.2003.00757.x 12790863

[47] Gallant MH Beaulieu MC Carnevale FA. Partnership: an analysis of the concept within the nurse–client relationship. J Adv Nurs. (2002) 40:149–57. 10.1046/j.1365-2648.2002.02357.x 12366644

[48] Morghen S Morandi A Guccione AA Bozzini M Guerini F Gatti R. The association between patient participation and functional gain following inpatient rehabilitation. Aging Clin Exp Res. (2017) 29:729–36. 10.1007/s40520-016-0625-3 27590904

[49] Tobiano G Marshall A Bucknall T Chaboyer W. Patient participation in nursing care on medical wards: an integrative review. Int J Nurs Stud. (2015) 52:1107–20. 10.1016/j.ijnurstu.2015.02.010 25769475

[50] Oxelmark L Ulin K Chaboyer W Bucknall T Ringdal M. Registered Nurses' experiences of patient participation in hospital care: supporting and hindering factors patient participation in care. Scand J Caring Sci. (2018) 32:612–21. 10.1111/scs.12486 28675925

[51] Moreno ME Durán MM Hernandez Á. Nursing care for adaptation. Nurs Sci Q. (2009) 22:67–73. 10.1177/0894318408327296 19176862

[52] Roy C. Research based on the Roy adaptation model: last 25 years. Nurs Sci Q. (2011) 24:312–20. 10.1177/0894318411419218 21975478

[53] Roy SC. An explication of the philosophical assumptions of the Roy adaptation model. Nurs Sci Q. (1988) 1:26–34. 3281068 10.1177/089431848800100108

